# *Smelowskia sunhangii* (Brassicaceae), a new species from China, with a re-evaluation of the *S. tibetica* complex

**DOI:** 10.3897/phytokeys.178.62922

**Published:** 2021-06-04

**Authors:** Xin-jian Zhang, Jun-Tong Chen, Ihsan A. Al-Shehbaz, Qun Liu, Li-Juan Li, Peng-Ju Liu, Xian-Han Huang, Tao Deng

**Affiliations:** 1 CAS Key Laboratory for Plant Diversity and Biogeography of East Asia, Kunming Institute of Botany, Chinese Academy of Sciences, Kunming 650201, Yunnan, China Kunming Institute of Botany, Chinese Academy of Sciences Kunming China; 2 University of Chinese Academy of Sciences, Beijing 100049, China University of Chinese Academy of Sciences Beijing China; 3 Missouri Botanical Garden, 4344 Shaw Blvd., St. Louis, Missouri 63110, USA Missouri Botanical Garden St. Louis United States of America; 4 School of Life Sciences, Yunnan Normal University, Kunming 650092, Yunnan, China Yunnan Normal University Kunming China; 5 CAS Key Laboratory of Plant Germplasm Enhancement and Specialty Agriculture, Wuhan Botanical Garden, Chinese Academy of Sciences, Wuhan 430074, Hubei, China Wuhan Botanical Garden, Chinese Academy of Sciences Wuhan China

**Keywords:** Brassicaceae, China, Cruciferae, new species, phylogeny, taxonomy

## Abstract

*Smelowskia sunhangii*, from Qinghai and Tibet (China), is described and illustrated. Morphological and molecular data indicate that *S. sunhangii* is closely related to *Smelowskia tibetica*, from which it is easily distinguished by the densely hirsute (vs. glabrous or sparsely pubescent), elliptic to ovate-lanceolate (vs. suborbicular, oblong, or lanceolate) fruits with undulate (vs. straight) margins. A re-evaluation of the widely distributed *S. tibetica* and related taxa is also provided.

## Introduction

The genus *Smelowskia* C.A.Mey. (Brassicaceae; Cruciferae) comprises 25 species distributed mainly in central and northeastern Asia, with fewer species in North America (Al-Shehbaz and Warwick 2006). The North American-Beringian taxa are believed to have originated from Asian ancestors (Carlsen et al. 2010). However, as in many other genera of the family, *Smelowskia* lacks unique synapomorphies (Warwick et al. 2004), though it is currently placed in the monogeneric tribe Smelowskieae (Al-Shehbaz et al. 2006; Al-Shehbaz 2012). Schulz (1936) and Appel and Al-Shehbaz (2003) considered the genera *Ermania* Cham. ex Botsch., *Gorodkovia* Botsch. & Karav., *Hedinia* Ostenf., *Redowskia* Cham. & Schltdl., *Sinosophiopsis* Al-Shehbaz, and *Sophiopsis* O.E. Schulz to be morphologically closely related to *Smelowskia*. Warwick et al. (2004) and Al-Shehbaz and Warwick (2006) incorporated all these genera into *Smelowskia* based on molecular phylogenetic and morphological evidences.

Nine species of *Smelowskia* are native to China (Al-Shehbaz and Warwick 2006; German and Chen 2009), and they are distributed mainly in the western parts of the country. In their most recent account for the *Flora of China*, these species were placed by Zhou et al. (2001) in the genera *Hedinia*, *Sinosophiopsis*, *Smelowskia*, and *Sophiopsis*. Of these, *H. tibetica* (Thomson) Lipsky is the most widely distributed (Bhutan, W and Himalayan China, India, Kyrgyzstan, Nepal, Tajikistan) and was recognized by these authors as most highly variable in fruit shape. Four new taxa were described by Zhou and An (1990) and He and An (1996) from Xinjiang province, including *H. lata* Xue L.He & C.H.An, *H. rotundata* C.H.An, *H. taxkargannica* G.L.Zhou & C.H.An, H. taxkargannica var. hejigensis G.L.Zhou & C.H.An, but these were reduced by Zhou et al. (2001) and Al-Shehbaz and Warwick (2006) to synonymy of *Smelowskia tibetica* (Thomson) Lipsky based on the examination of all type collections of *Smelowskia*. The above *Smelowskia tibetica* complex is re-examined here in light of additional field and molecular studies.

**Figure 1.**
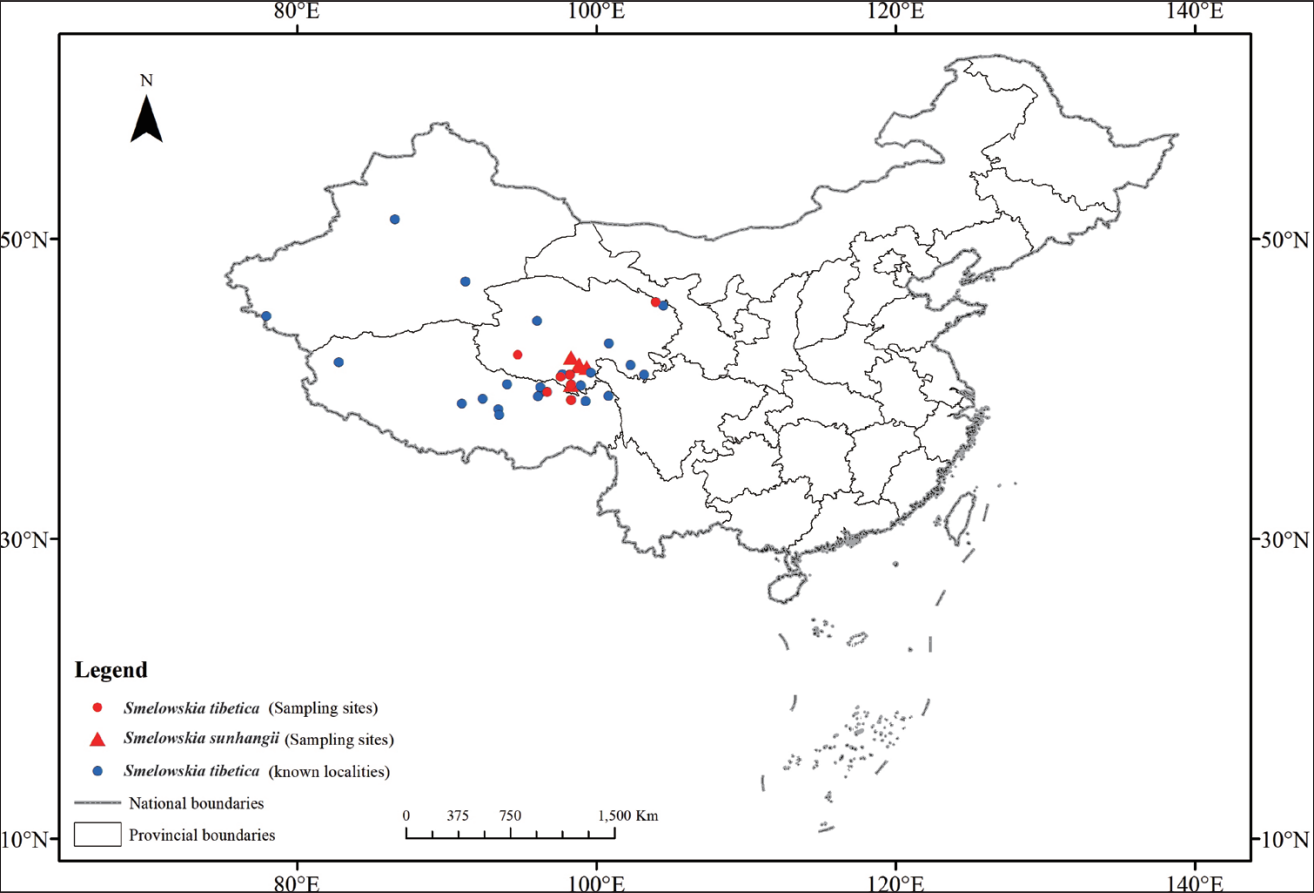
Distributions of sampling sites (red triangles) of *Smelowskia sunhangii*, known localities (blue dots) and sampling sites (red dots) of *Smelowskia tibetica* in China.

During a recent field survey, we collected an unidentified specimen of *Smelowskia* in Qinghai province, China (Fig. 1), and it appeared initially to be allied morphologically to the *S. tibetica* complex. The current study is devoted to resolve the mystery of the Chinese components of this complex.

## Materials and methods

### Morphological observation

Morphological data were recorded from field collections and herbarium specimens covering the full spectrum of geographical, plant type, and habitat variation in the *S. tibetica* complex. The voucher specimens of our collections were deposited in the herbarium of Kunming Institute of Botany (**KUN**), Kunming, China. Herbarium specimens of the *S. tibetica* complex and related taxa were examined from BNU, KUN, PE, WUK and XJA (acronyms follow Thiers 2018), either by examining the specimens directly, or electronically through the National Plant Specimen Resource Center (http://www.cvh.ac.cn/index.php), and JSTOR Global Plants web portal (https://plants.jstor.org/). The voucher specimens for morphological observation were cited in the section of “Chinese specimens examined” of taxonomic treatment and Table 1.

**Table 1.** Voucher information and GenBank accessions for phylogenetic analysis.

Taxon	Voucher	GenBank accession number
*Smelowskia tibetica* (1) (as *Hedinia rotundata*)	*Yang Jingsheng 402* (KUN)	MZ089467
*S. tibetica* (2) (as *Hedinia lata*)	*HuangXH018-9* (KUN)	MZ089476
*S. tibetica* (4) (as *H. lata*)	*HuangXH015-20* (KUN)	MZ089475
*S. tibetica* (5) (as *H. lata*)	*Deng7359* (KUN)	MZ089474
*S. tibetica* (6) (as *H. tibetica*)	*ZDG23-7* (KUN)	MZ089468
*S. tibetica* (7) (as *H. tibetica*)	*Deng7261* (KUN)	MZ089473
*S. sunhangii* (1)	*Deng7262* (KUN)	MZ089472
*S. sunhangii* (3)	*DengT128-9* (KUN)	MZ089471
*S. sunhangii* (4)	*HuangXH025-13* (KUN)	MZ089470
*S. sunhangii* (5)	*DengT105-23* (KUN)	MZ089469
*Shehbazia tibetica*	*HuangXH028-4* (KUN)	MZ089466
**Sequences downloaded from NCBI**		
*Smelowskia sunhangii* (2) (as *S. tibetica*)	*Zh641* (KUN)	KX244397
*S. tibetica* (3) (as *H. lata*)	*LJQ-QLS-2008-0115* (KUN)	JF941772
*S. czukotica*	*TC03_60*	EU489520
*S. altaica*	*TC03_61*	EU489519
*S. bartholomewii*	*Ho et al. 3000* (MO)	AY230609
*S. sophiifolia*	*Geonova 148* (LE)	AY230608
*S. calycina*	*Velychnin N495* (LE)	AY230604
*S. borealis*	*Murray 8582* (DAO)	AY230571
*S. jacutica*	*Elias & Murray 11462* (ALA)	AY230646
*S. johnsonii*	*Johnson*, *Viereck*, *& Melchior 688* (ALA)	AY230631
*S. ovalis*	*CCDB-23367-G06*	MG234816
*S. sisymbrioides*	*Egorova 2349* (LE)	AY230612
*S. annua*	*Anonymous 870473* (HNWP)	AY230610
*Descurainia pinnata*	–	AF183122
*Shehbazia tibetica* (as *Dontostemon tibeticus*)	*GH:33576*	LN713849

### Molecular analyses

We sampled 12 collections representing the *Smelowskia tibetica* complex, including the presumed new species. Leaf materials were collected from field works and dried herbarium specimens. *Descurainia pinnata* from tribe Descurainieae (sister to tribe Smelowskieae) and *Shehbazia tibetica* from tribe Chorisporeae were selected as outgroups based on previous molecular phylogenetic relationships (Warwick et al. 2004; Al-Shehbaz et al. 2006; Al-Shehbaz 2012; Liu et al. 2020). Sequences for other taxa were obtained from GenBank (Table 1). Voucher information and GenBank accession numbers are also provided in Table 1.

Total genomic DNA extracted from leaf materials using DP305 Plant Genomic DNA kits (Tiangen, Beijing, China) following the manufacturer’s protocol. The entire ITS region (including internal transcribed spacers ITS1 and ITS2 of nuclear ribosomal DNA and the 5.8S rRNA gene) were amplified using the primers ITS1-18S described in O’Kane et al. (1997) and ITS4 described in White et al. (1990). Parallel chromatograms derived from bi-directional sequencing were checked for accuracy by visual inspection with Chromas v. 2.6.6 (http://www.technelysium.com.au) and integrated into a single sequence. Sequences were then aligned with MEGA version 7.0 and gaps were treated as missing data (Sudhir et al. 2016).

Phylogenetic reconstruction was performed using Bayesian inference (BI) and maximum likelihood (ML). The phylogenetic tree based on Bayesian inference was generated using MrBayes version 3.2.6 (Huelsenbeck and Ronquist 2001). The phylogenetic analysis based on maximum likelihood was conducted with PhyML version 3.0 (Guindon et al. 2010). Detected by the jModeltest 2.1.7, the GTR+G model selected by Akaike information criterion (AIC) was used in BI and ML analyses (Posada 2008).

## Results

### Morphology and taxonomy

Morphological studies of strictly Chinese material revealed a wide variation in fruit indumentum and shape and leaf divisions in the *Smelowskia tibetica* complex, and such differences lead Zhou and An (1990) and He and An (1996) to recognize several novelties under the synonymized *Hedinia*. For example, plants with 1- or 2-pinnatifid leaves and oblong glabrous fruits characterize the type collection of *Smelowskia tibetica*, those with 2-pinnatifid leaves and glabrous oblong fruits are found in the type of *H. lata*, those with lanceolate glabrous fruits are in plants of the type of *H. taxkargannica*, and those with pubescent suborbicular fruits are seen in the type of *H. rotundata*. All of the above taxa do not resemble the densely fruited plants we collected in Qinghai (Table 2).

**Table 2.** Comparison of selected distinguishing features of *Smelowskia sunhangii* and related taxa.

Taxon	Cauline leaf shape	Fruit shape	Indumentum in silicle	Distribution
*Smelowskia tibetica*	1- or 2-pinnatifid	oblong	Glabrous	Qinghai, Tibet, Xinjiang
*S. tibetica* (as *Hedinia lata*)	2-pinnatifid	oblong	Glabrous	Qinghai, Tibet, Xinjiang
*S. tibetica* (as *H. taxkargannica*)	1-or 2-pinnatifid	lanceolate	Glabrous	Xinjiang
*S. tibetica* (as *H. rotundata*)	1-pinnatifid	suborbicular	sparsely pubescent	Xinjiang
*S. sunhangii*	1-pinnatifid	elliptic to ovate-lanceolate	densely hirsute	Qinghai, Tibet

These densely hirsute plants with densely hirsute, elliptic to ovate-lanceolate fruits undulate along the margin, do not match any of the other 25 species we carefully studied throughout the range of *Smelowskia*. Therefore, these Qinghai plants are described as the new species *S. sunhangii* and recognized hereafter as such.

### Phylogenetic analyses

**Figure 2.**
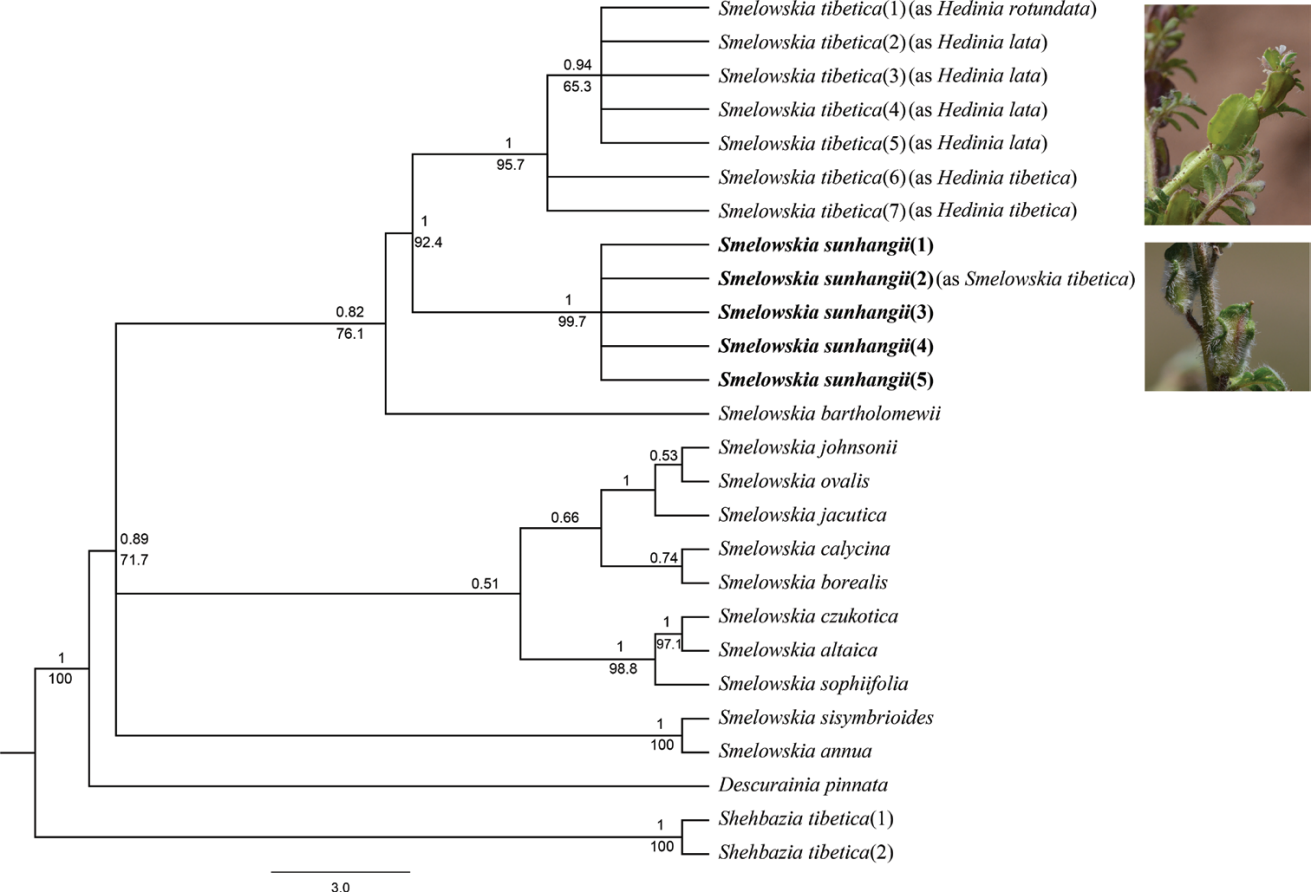
Bayesian consensus tree of 13 species of *Smelowskia* based on their ITS sequences, with *Descurainia pinnata* and *Shehbazia tibetica* as the outgroup. Numbers above branches indicate Bayesian posterior probability, numbers below branches are ML bootstraps.

A total of 15 taxa were included in this analysis (Fig. 2). The resulting multiple alignment of the ITS region, including 5.8S gene, was 651 bp. The 50% majority-rule consensus tree based on Bayesian posterior probability (PP) and maximum likelihood bootstraps (ML) of the ITS sequences both showed that four accessions of *Smelowskia sunhangii* grouped together (PP=1, ML=99.7), however, an accession of previously determined *Smelowskia tibetica* (KX244397) was nested in it. After a critical examination of the voucher of this accession, *Zh641* (KUN), we immediately concluded that it is *S. sunhagii*.

All of the seven accessions of the *Smelowskia tibetica* complex formed a monophyletic clade, sister to *S. sunhangii* with a strong support (PP = 1, ML = 95.7). In addition, the clade of *Smelowskia tibetica* complex formed a polytomy of three subclades, of which two belong to *Smelowskia tibetica* and the third is a polytomy of samples identifiable as members of the *Hedinia lata* and *H. rotundata* as characterized morphologically above.

## Discussion

Our molecular and morphological analyses indicate that *Smelowskia tibeticasensu lato* contains two, easily distinguished species, of which one is the new *S. sunhangii* and the other is *S. tibetica* including the taxa described by Zhou and An (1990) and He and An (1996) as three species and one variety of *Hedinia*. Although our data show some differentiation within *S. tibetica*, namely a polytomy of the *Hedinia* taxa forming another polytomy with the remaining samples of *S. tibetica* proper, such a current slight differentiation does not justify the recognition of more than two species. The principal reasons for not recognizing the *Hedinia* taxa are as follows.

First and foremost, as discussed above in the section of “Morphology and taxonomy” of the results, the variation in leaf and fruit morphology in the entire *Smelowskia tibetica* complex was critically studied by one of us (Al-Shehbaz) in plants from the entire range of this complex in Bhutan, India, China, Nepal, and Tajikistan both in herbaria worldwide and in the field in Xinjiang (China), Nepal, and Tajikistan. The conclusion of such observations was the acceptance of a single polymorphic species in the Himalayan region (Al-Shehbaz 2015), including Nepal and its neighbors (Al-Shehbaz and Watson 2011). That conclusion was also reflected in BrassiBase (Kiefer et al. 2014), the comprehensive and continuously updated database on the entire Brassicaceae.

Then similar variation in fruit and leaf morphology of the *Smelowskia tibetica* complex, especially that of the *Hedinia* taxa discussed above, was observed sporadically elsewhere in the species range and, therefore, the alleged distinction of *Hedinia* taxa has no merit.

Additionally, although our molecular data show a slight differentiation in the *Hedinia* taxa (Fig. 2), our sampling cannot be considered as the final word without doing similar analyses from the other countries where *Smelowskia tibetica* grows. We only used just two samples of this highly variable and widespread species. Therefore, the most reasonable conclusion is to avoid the recognition of any of the *Hedinia* taxa and create additional synonymies without more convincing data.

Finally, our morphological studies strongly support the novelty of *Smelowskia sunhangii* because its fruit morphology in unique in the genus and has not yet been observed in Asian and North American taxa. Furthermore, our molecular data also strongly support the above recognition of the novelty and its sister relationship to *S. tibetica* including the *Hedinia* taxa of Zhou and An (1990) and He and An (1996).

### Taxonomic treatment

#### Smelowskia sunhangii

Taxon classificationPlantaeBrassicalesBrassicaceae

T. Deng, X.J. Zhang & J.T. Chen sp. nov.

5E74A862-62F1-55E7-B7A4-E6B5A9DD5B4E

urn:lsid:ipni.org:names:77217427-1

Figs 3 , 4

##### Type.

China. Qinghai. Yushu Tibetan Autonomous Prefecture, Yushu City, Longbao Town, 33°30'13.54"N, 96°24'38.79"E, 4,602 m, 26 July 2019, *J.T. Chen*, *X. Zhang & T.H. Kuang HuangXH025-13* (holotype: KUN1498313!; isotype: KUN1498314!).

##### Diagnosis.

*Smelowskia sunhangii* is easily distinguished from its closest relative *S. tibetica* by having densely hirsute (vs. glabrous or sparsely pubescent), elliptic to ovate-lanceolate (vs. oblong, suborbicular, or lanceolate) fruits undulate (vs. straight) along margin.

**Figure 3.**
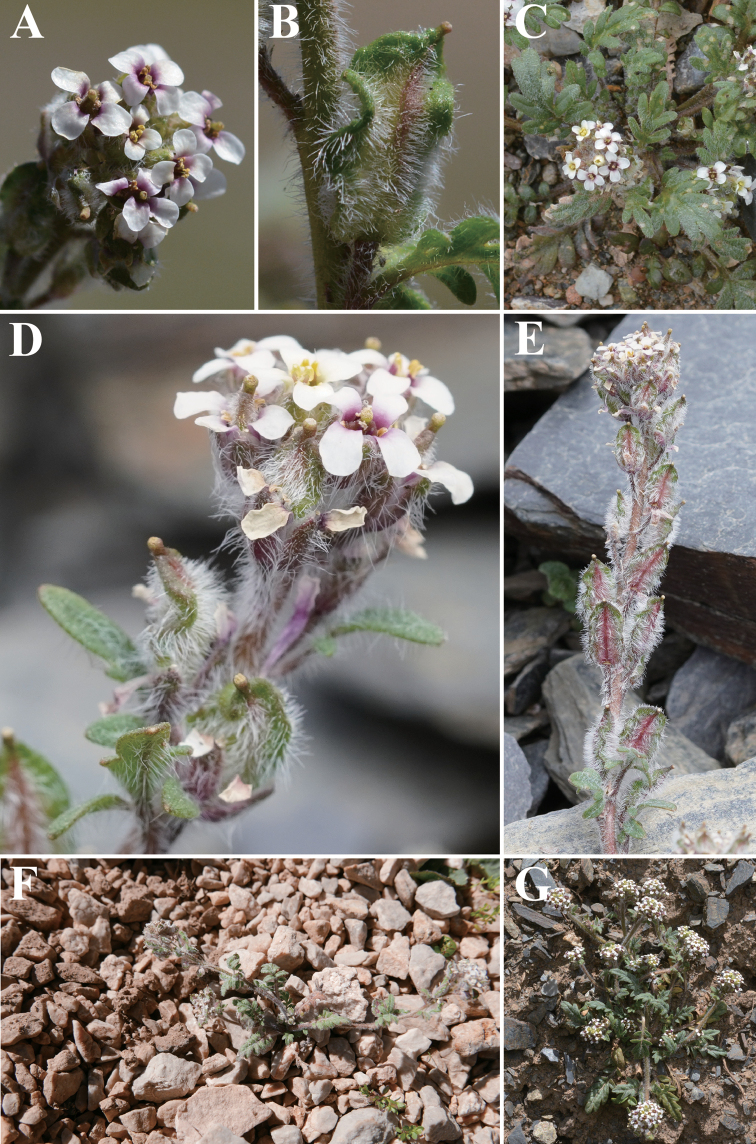
*Smelowskia sunhangii* T. Deng, X.J. Zhang & J.T. Chen **A** flower **B** fruit **C** leaves and flowers **D** inflorescence **E** infructescence **F, G** plants and habitat.

##### Description.

Herbs 5–15 cm tall, covered with simple trichomes, canescent. Stems procumbent or ascending, densely white hirsute. Basal leaves densely hirsute; petiole 0.5–1.5 cm long, often ciliate basally; leaf blade ovate or narrowly oblong in outline, 1-pinnatifid, 1–4 × 1–2.5 cm; cauline leaves similar to basal, reduced in size upwards. Racemes bracteate throughout, distal bracts subsessile. Sepals oblong, 1–2 × 0.5–0.8 mm, hirsute. Petals obovate, 2–3.2 × 0.9–1.4 mm, claw ca. 1.5 mm long. Fruit elliptic to ovate-lanceolate, densely white hirsute, 5–10 × 3–5 mm, undulate along margin, appressed to rachis. Seeds light to dark brown, oblong, 0.9–1.2 × 0.4–0.6 mm. Fl. Jul–Sep, fr. Aug–Oct.

##### Etymology.

*Smelowskia sunhangii* is named after Prof. Sun Hang (1963–), director of the Kunming Institute of Botany (China) who conducted extensive research on plant taxonomy, biogeography, and evolutionary biology and made outstanding contributions towards understanding the plant diversity of China. Vernacular name: The Chinese name is given as “毛果藏荠” (máo guǒ zàng jì), referring to the densely hirsute fruit of the new species.

**Figure 4.**
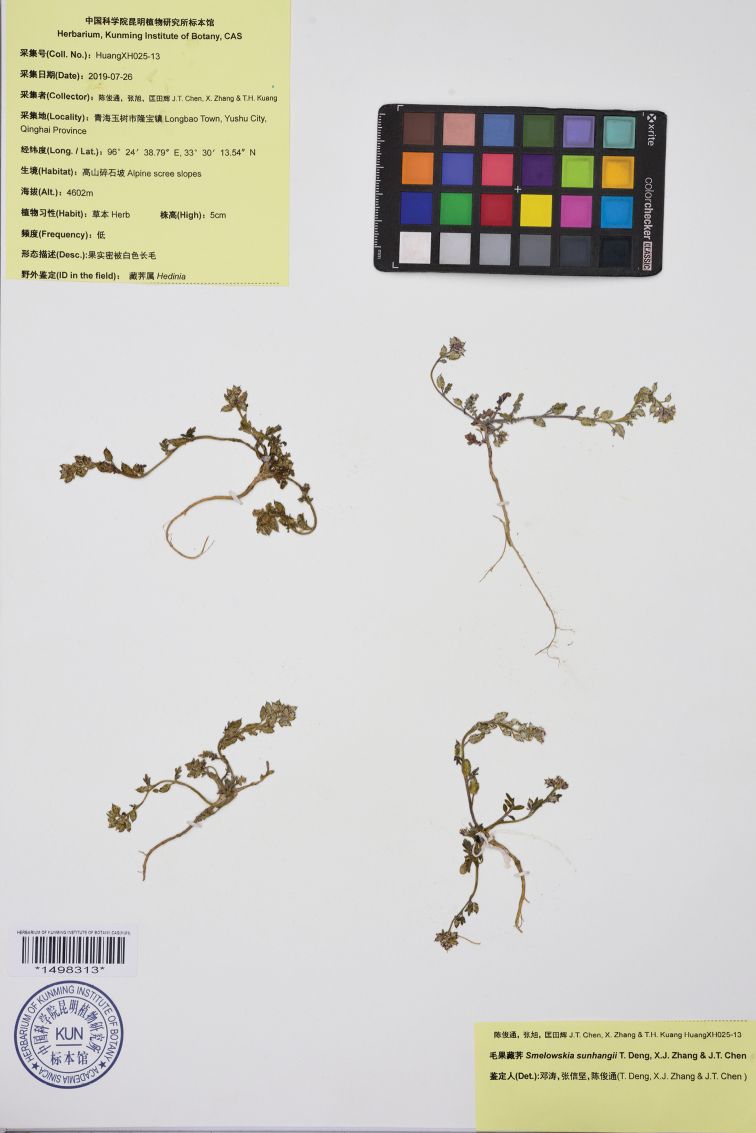
Photograph of the holotype of *Smelowskia sunhangii* T. Deng, X.J. Zhang & J.T. Chen (KUN1498313).

##### Distribution.

China (Qinghai, Tibet [Xizang], Fig. 1).

##### Paratypes.

China. **Tibet**: Lhasa, Damxung County, Gangla Mountain, 30°41'6.77"N, 91°6'16.88"E, 4,802 m, 27 July 2018, *D.G. Zhang DengT051-14* (KUN). **Qinghai**: Yushu Tibetan Autonomous Prefecture, Yushu City, 32°17'20.62"N, 95°50'15.29"E, 4,848 m, 23 July 2019, *P.J. Liu & H.H. Shi deng7262* (KUN); Yushu Tibetan Autonomous Prefecture, Chindu City, 31 July 2019, *T. Deng*, *X.H. Huang*, *Z.Y. Lv & L.J. Li DengT128-9* (KUN); Yushu Tibetan Autonomous Prefecture, *T. Deng*, *X.H. Huang*, *Z.Y. Lv & L.J. Li DengT105-23* (KUN); Yushu Tibetan Autonomous Prefecture, Qumarlêb County, 2 September 2013, *J.W. Zhang*, *B. Yang & H.L. Chen Zh641* (KUN).

#### Smelowskia tibetica

Taxon classificationPlantaeBrassicalesBrassicaceae

(Thomson) Lipsky

68E4D6FA-2E1C-5148-9CB6-C44E0E13BC06

Basionym: Hutchinsia tibetica Thomson, Hooker’s Icon. Pl. 9: t. 900. 1852. TYPE: WESTERN TIBET. Lanak Pass, 18–19,000 ft [ca. 5,480–5,790 m], *Thomas Thomson s.n.* (holotype: K!).
Synonyms: Hedinia tibetica (Thomson) Ostenf. in Hedin, S. Tibet 6: fig. 2. 1922.
Hedinia lata Xue L.He & C.H.An, Acta Phytotax. Sin. 34(2): 205. 1996. TYPE: TIBET. Ando, alt. 4,750 m, on stony mountain slopes, 14 August 1963, *J.X. Yang 2220* (holotype: WUK, n.v.).
Hedinia rotundata C.H.An, *Acta Bot. Boreali-Occid. Sinica* 10: 325. 1990. TYPE: XINJIANG. The west of Altum Mountains, Qimantag, 2,700 m, 25 July 1984, *Zhang Li-Yun 84-A-411* (holotype: XJA!)
Hedinia taxkargannica G.L.Zhou & C.H.An, Acta Bot. Boreal.-Occid. Sin. 10: 323. 1990. TYPE: XINJIANG. Tajik Autonomous County of Taxkargan Vaka, in alpina desert steppe zone, July 1986, *An Zheng-xi N. 268* (holotype: XJA!)

##### Description.

Detailed descriptions of the species are found in the Flora of China (Zhou et al. 2001), Nepal (Al-Shehbaz and Watson 2011), and the entire Himalayan Region (Al-Shehbaz 2015). Therefore, there is no need to repeat it here.

##### Distribution.

China (Gansu, Qinghai, Sichuan, Tibet, Xinjiang, Fig. 1), Bhutan, India, Kyrgyzstan, Nepal, Tajikistan.

##### Chinese specimens examined.

China. **Tibet**: Baingoin County, 15 August 1988, *S.G. Wu*, *H. Ohba*, *Y.H. Wu & Y. Fei No. 4095* (KUN); Lhasa, Damxung County, Namtso, 25 July 2018, *D.G. Zhang*, *Y. Wu & H. Ye ZDG18-9* (KUN); Nagqu, Sog County, 28 July 2018, *D.G. Zhang*, *Y. Wu & H. Ye ZDG23-7* (KUN); Nagqu, Sog County, 28 July 2018, *D.G. Zhang*, *Y. Wu & H. Ye ZDG24-24* (KUN); Nagqu, Shenza County, 3 August 1987, *B.S. Li & D. Zheng 10888* (PE); Nagqu, Biru County, 3 September 1976, *D.D. Tao 11181a* (KUN); Nagqu, Anduo County, 10 September 2008, *J.H. Chen*, *H.F. Zhuang & D.T. Liu Yangyp-Q-0258* (KUN); Nagqu, Baqing County, 26 June 2016, *J.P. Yue*, *Z. Zhou & H.L. Chen YZC226* (KUN); Nagqu, Anduo County, 21 August 2009, *J.H. Chen*, *H.F. Zhuang & P. Tashi YangYP-Q-2166* (KUN); Ngari, Rutog County, 29 July 1987, *B.S. Li*, *D. Zheng 10848* (PE); Changdu, Jomda County, 1 August 2004, *D.E. Boufford*, *J.H. Chen*, *S.L. Kelley*, *J.Li*, *R. H. Ree*, *H. Sun*, *J.P. Yue & Y.H. Zhang 31531* (KUN); Changdu, Riwoqê County, 11 August 2004, *D.E. Boufford*, *J.H. Chen*, *S.L. Kelley*, *J. Li*, *R.H. Ree*, *H. Sun*, *J.P. Yue & Y.H. Zhang 32037* (KUN). **Qinghai**: Yushu Tibetan Autonomous Prefecture, Nangqên County, 24 July 1965, *Y.C. Yang 1261* (KUN); Yushu Tibetan Autonomous Prefecture, Nangqên County, 24 July 2019, *X. Zhang*, *J.T. Chen & T.H. Kuang HuangXH018-9* (KUN); Yushu Tibetan Autonomous Prefecture, Nangqên County, 23 July 2019, *X. Zhang*, *J.T. Chen & T.H. Kuang HuangXH015-20* (KUN); Yushu Tibetan Autonomous Prefecture, Yushu City, 23 July 2019, *P.J. Liu & H.H. Shi deng7261* (KUN); Yushu Tibetan Autonomous Prefecture, Zadoi County, 25 July 2019, *P.J. Liu & H.H. Shi deng7359* (KUN); Haibei Tibetan Autonomous Prefecture, Menyuan Hui Autonomous County, 26 July 2008, *Y.H. Wu LJQ-QLS-2008-0115* (KUN); Haibei Tibetan Autonomous Prefecture, Menyuan Hui Autonomous County, 18 June 2018, *X.X. Zhu & Y.M. An ZXX18140* (KUN); Golog Tibetan Autonomous Prefecture, Madoi County, 31 July 2011, *S.L. Chen*, *Q.B. Gao & F.Q. Zhang ChenSL1379* (KUN); Golog Tibetan Autonomous Prefecture, Baima County, 29 August 2013, *J.W. Zhang*, *B. Yang & H.L. Cheng Zh537* (KUN); Golog Tibetan Autonomous Prefecture, Darlag County, 10 August 1993, *H.B.G. 1033* (PE); Haixi Mongol and Tibetan Autonomous Prefecture, Golmud City, 11 September 2008, *H.Y. Feng LiuJQ-08KLS-139* (KUN); Haixi Mongol and Tibetan Autonomous Prefecture, Golmud City, 19 August 2010, *X.M. Tian*, *Z.Q. Wang & J.B. Zou LiuJQ-Txm10-097* (KUN); Haixi Mongol and Tibetan Autonomous Prefecture, Golmud City, Fenghuo mountain, 18 August 2010, *X.M. Tian*, *Z.Q. Wang & J.B. Zou Liujq-Txm10-074* (KUN); Haixi Mongol and Tibetan Autonomous Prefecture, Golmud City, Ulan Moron, 22 August 1990, *Yang Jingsheng 402* (KUN). **Xinjiang**: Bayin’gholin Mongol Autonomous Prefecture, Ruoqiang County, 10 August 1988, *S.G. Wu*, *H. Ohba*, *Y.H. Wu & Y. Fei 2678* (KUN); Bayin’gholin Mongol Autonomous Prefecture, Hejing County, 16 August 1958, *J.N. Zhu & A.R. Li 6543* (KUN); Kashgar Prefecture, Taxkorgan Tajik Autonomous County, 11 August 2008, *J. Qiu & J.J. Feng LiuJQ0171* (KUN).

## Supplementary Material

XML Treatment for Smelowskia sunhangii

XML Treatment for Smelowskia tibetica
